# Balance ability and all-cause death in middle-aged and older adults: A prospective cohort study

**DOI:** 10.3389/fpubh.2022.1039522

**Published:** 2023-01-09

**Authors:** Kaihong Xie, Xiao Han, Xuanhan Hu

**Affiliations:** ^1^School of Nursing, Zhejiang Chinese Medical University, Hangzhou, China; ^2^School of Health Humanities, Peking University Health Science Center, Beijing, China; ^3^The Second School of Clinical Medicine, Zhejiang Chinese Medical University, Hangzhou, China

**Keywords:** balance ability, death, overweight, obesity, chronic disease, elderly

## Abstract

**Objective:**

The present study aimed to explore the relationship between balance ability and all-cause death in middle-aged and elderly people and to provide a basis for formulating a balanced training plan for middle-aged and older people in China.

**Methods:**

Based on data from the China Health and Retirement Longitudinal Study (CHARLS) carried out in the years 2011, 2013, 2015, and 2018, 18,888 participants aged 45 years and above were included. Cox proportional hazard models were designed to evaluate the effect of balance ability on death events.

**Results:**

The present study found that there was an association between balance ability and death among middle-aged and older people. Multivariate Cox proportional hazard regression model analysis showed that the risk of death decreased by 10% (*HR* = 0.90,95% CI: 0.85–0.95) for every second increase in balance ability. With balance ability <10 s as the reference group, the adjusted *HRs* were 0.61 (0.44–0.85) among middle-aged and elderly people. The death density of balance ability of <10 s was 73.87 per thousand person-years higher than that of ≥10 s. There was no interaction between balance ability and chronic disease, overweight, and obesity (*P* > 0.05).

**Conclusion:**

The risk of all-cause death in middle-aged and older people increased with the decrease in balance ability and showed no statistical significance between chronic disease, overweight, and obesity, as corroborated by the present study.

## 1. Introduction

Balance ability is a complex body function that is coordinated by multiple systems, and the peak value decreases with age in adults (1). A variety of causes, such as somatosensory imbalance (2), brain damage (3), spinal cord injury (4), and decreased skeletal muscle mass (5), lead to impaired balance in the elderly. Chronic non-communicable diseases are referred to as chronic diseases, and mainly refer to hypertension, diabetes, stroke, etc., (6). It is predicted that, by 2030, death from chronic diseases would increase to 62.7% by way of lost tax revenue and 51.6% by way of additional payment benefits (7). Being overweight and obese are both chronic diseases and major causes of other chronic diseases (8). Chronic diseases have an impact on the relationship between balance ability and death (9). Previous studies on balance ability have focused on the elderly population aged 51–75 years (9, 10) and were mainly focused on the relationship with falls (11, 12). Studies showed that the decline in physical function begins early in life (13), and more and more studies are using physical indicators as predictors of death (14). It has been indicated that successful 10-s one-legged stance (10-s OLS) performance might be able to predict survival (9). Balance ability is a low-cost screening tool to prevent adverse outcomes (1). However, participants with vestibular disease were unable to perform 10-s OLS and those with decreased muscle strength in both legs were limited. The present study uses the balance ability developed by the China Health and Retirement Longitudinal Study (CHARLS), a semi-tandem stand test, to explore the association between balance ability and death. The study aimed to provide a reference for early intervention of balance ability in middle-aged and older people and improve their adverse outcomes.

## 2. Manuscript formatting

### 2.1. Ethics statement

The Ethical Review Committee at Peking University (IRB 00001052-11014) approved CHARLS for the biomarker sample collection.

### 2.2. Study population

**Figure 1:**
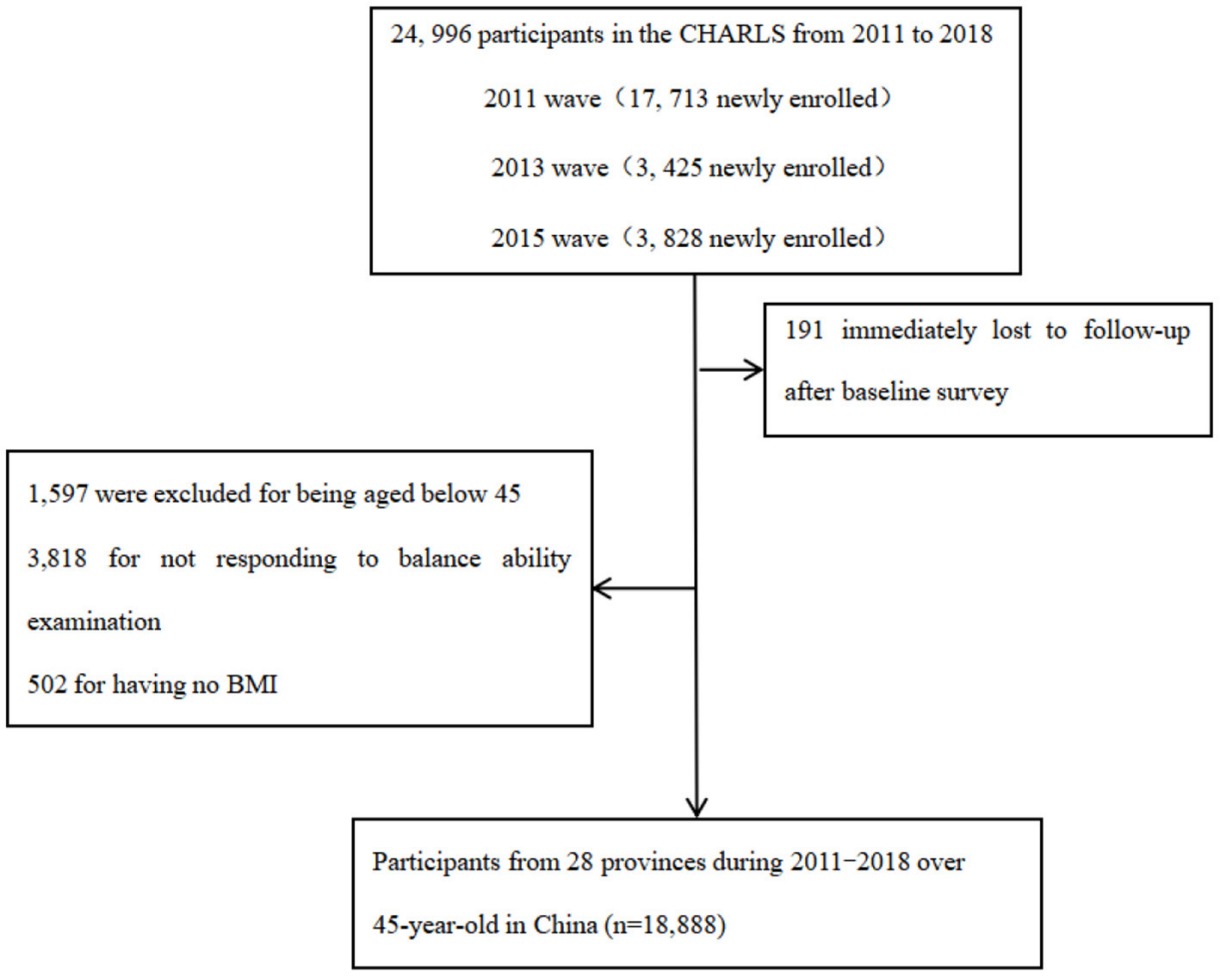
Flowchart of sample selection.

This study includes 24,805 participants who were 45 years of age and above as per the CHARLS of 2011–2018. In Figure 1, this study also excluded participants below the age of 45 years from all analyses (*n* = 1,597). Participants who did not have balance ability measures (*n* = 3,818) for that year were excluded, resulting in a final sample size of 19,390 participants. Additionally, participants who did not have body mass index (BMI) measures were excluded (*n* = 512).

### 2.3. Exposure

Interviewers measured balance ability, which refers to the number of seconds in which a participant can semi-tandem stand for 10 s without moving or holding anything. If the participant could semi-tandem stand for 10 s, then 10 s was recorded (15, 16). Otherwise, specific seconds were recorded, which is a continuous variable. The present study generates binary variables based on the median of balance ability.

### 2.4. Outcome

The death information for each participant and the outcome variable were obtained from death registration and certification by asking their relatives or community managers in 2013, 2015, and 2018, or at the end of follow-up (March 31, 2019). Death events were identified as “1” and survival and censorship as “0.” The survival time was calculated according to the outcome (censored participations' survey year counted as survival time).

### 2.5. Covariates

According to our conceptual framework, low socioeconomic position and health status are strong determinants of all-cause death. Sociodemographic status includes age, gender, marital status, *hukou*, and education. Education is classified into five categories (no formal education, primary school, middle or high school, and college or above). The term *hukou* includes agricultural *hukou* and non-agricultural *hukou*. Health-related factors include self-reported smoking, drinking alcohol (never, former, or current), self-reported diseases (hypertension, diabetes, cancer, stroke, and memory diseases), activities of daily living (AOLs) / instrumental activities of daily living (ADLs/IADLs), and body mass index (BMI).

### 2.6. Statistical analysis

For summary statistics, the present study employed means and standard deviation (SDs) to describe continuous variables conforming to the hypothesis of normal distribution. Categorical variables were described by the frequency with percentage. Based on the baseline characteristics of balance ability, we deemed the chi-square (χ^2^ test), analysis of variance (ANOVA), or Mann–Whitney *U*-test as appropriate. Assuming missing at random, incomplete observations were imputed with multivariate imputation *via* classification or regression trees. Ten imputed data sets were generated and pooled using R 4.2.1.

To examine the association between balance ability and death events of all causes, Cox proportional hazards models were used to calculate hazard ratios (*HR*s) with 95% confidence intervals (CIs). The risk of death was calculated based on a specific balance. Three models were designed and compared for middle-aged and elderly people. Model 1 was adjusted for age and gender. Model 2 is based on Model 1 with supplementary adjustment of marital status, household registration, education, smoking, drinking, and BMI. All covariates (15 covariables) were included in Model 3. Chronic disease status and overweight and obesity were analyzed interactively between balance ability and death to study their effects. An interaction analysis was performed to examine the potential association between balance ability and death and to determine whether the results were regulated by chronic diseases, overweight, and obesity. Finally, we performed the sensitivity analysis by repeating all analyses using an original data set without multiple interpolations.

## 3. Results

### 3.1. Patient characteristics

A total of 18,888 eligible middle-aged and older people took part in this study, including 9,226 men and 9,650 women, with 57.9 ± 9.6 years of age (Figure 1). During the follow-up, 1,329 (7.0%) middle-aged and older people had died, and the median follow-up time was 7 years (Table 1). In total, there were 10,256 participants with non-overweight and obesity and 8,632 participants with overweight and obesity. There were 7,554 participants with a non-chronic health condition and 11,334 participants with chronic diseases.

**Table 1:** Baseline characteristics of 18,888 participants according to death.

**Baseline characteristics**	**Total sample (*n* = 18,888)**	**Survival (*n* = 17,559)**	**Death (*n* = 1,329)**	***P***	***SMD***
**Age, mean (SD)**	57.86 ± 9.60	57.14 (9.14)	67.34 (10.52)	<0.001	1.035
**Gender**, ***n*** **(%)**				<0.001	0.301
Men	9 226 (48.9)	8,394 (47.8)	832 (62.7)		
Women	9 650 (51.1)	9,154 (52.2)	496 (37.3)		
BMI, mean (SD)	23.9()	23.98 (3.97)	22.17 (4.14)	<0.001	0.445
**Married**, ***n*** **(%)**				<0.001	0.397
No	16 739 (88.6)	15,745 (89.7)	994 (74.8)		
Yes	2 149 (11.4)	1,814 (10.3)	335 (25.2)		
***hukou***, ***n*** **(%)**				<0.001	0.122
Agricultural	13,745 (78.5)	1,2667 (78.1)	1078 (82.9)		
Non-agricultural	3,775 (21.5)	3,553 (21.9)	222 (17.1)		
**Education**				<0.001	0.363
No formal education	7,789 (44.0)	7,037 (42.9)	752 (57.7)		
Primary school	3,936 (22.2)	3,642 (22.2)	294 (22.6)		
Middle or high school	5,572 (31.5)	5,333 (32.5)	239 (18.3)		
College or above	395 (2.2)	377 (2.3)	18 (1.4)		
**Smoking**, ***n*** **(%)**				<0.001	0.352
Never	6,032 (32.0)	5,511 (31.4)	521 (39.3)		
Formal	1,547(8.2)	1,339 (7.6)	208 (15.7)		
Current	11,279(59.8)	10,681 (60.9)	598 (45.1)		
**Drinking**, ***n*** **(%)**				0.004	0.101
Never	5,037 (26.7)	4,657 (26.6)	380 (28.7)		
Formal	1,624 (8.6)	1,541 (8.8)	83 (6.3)		
Current	12,194 (64.7)	11,331 (64.6)	863 (65.1)		
**History of**					
**comorbidities**, ***n*** **(%)**					
Hypertension	3,931 (20.8)	3,517 (20.0)	414 (31.2)	<0.001	0.257
Diabetes	971 (5.1)	855 (4.9)	116 (8.7)	<0.001	0.154
Stroke	301 (1.6)	252 (1.4)	49 (3.7)	<0.001	0.143
Cancer	186 (1.0)	157 (0.9)	29 (2.2)	<0.001	0.105
Memory disease	203 (1.1)	168 (1.0)	35 (2.6)	<0.001	0.127
**ADL**, ***n*** **(%)**				<0.001	0.360
No	16,752 (88.7)	15,738 (89.6)	1,014 (76.3)		
Yes	2,136 (11.3)	1,821 (10.4)	315 (23.7)		
**IADL**, ***n*** **(%)**				<0.001	0.399
No	15,824 (83.8)	14,915 (84.9)	909 (68.4)		
Yes	3,064 (16.2)	2,644 (15.1)	420 (31.6)		
No	12,432 (65.8)	11,538 (65.7)	894 (67.3)		
Yes	6,456 (34.2)	6,021 (34.3)	435 (32.7)		
Balance ability, mean (SD)	10.0 (0.5)	9.97 (0.41)	9.85 (0.96)	<0.001	0.165
**Balance ability**, ***n*** **(%)**				<0.001	0.171
<10 s	156 (1.0)	117 (0.7)	39 (2.9)		
≥10 s	18,732 (99.0)	17,442 (99.3)	1,290 (97.1)		

SD, standard deviation. (A) *P*-values are based on chi-square (χ^2^) or analysis of variance (ANOVA) or Mann-Whitney *U*-test. (B) Measured in the subpopulation of 18,888 participants. (C) Missing data instances: 12 for gender, 1,196 for educational level, 30 for smoking, 33 for drinking, and 1,368 for *hukou*.

### 3.2. Multivariate Cox proportional hazards regression models between balance ability and death risk

After adjusting for confounding factors such as demography and health behavior, the risk of death decreased by 10% (HR = 0.90,95% CI: 0.85–0.95) for every second increase in balance ability. After adjusting for confounding factors, the risk of death in middle-aged and older people with balance ability ≥10 s was reduced by 40% (HR = 0.61,95% CI: 0.44–0.85). The death density of middle-aged and older people with a balance ability of <10 s was 73.87 per thousand person-years higher than those ≥10 s (Table 2).

**Table 2:** Construction of Cox proportional hazards model for balance ability to death.

**Outcomes death of all causes**		**Incidence rate, per 1,000 person-years**	***HR*** **(95% CI)**		
	**Cases, no**		**Model 1**	**Model 2**	**Model 3**
**Balance ability (continuous)**	18,888	27.06	0.88 (0.84–0.93)^*^	0.89 (0.84–0.94)^*^	0.90 (0.85–0.95)^*^
**Balance ability (binary)**					
<10 s	156	100.32	1.00	1.00	1.00
≥10 s	18,732	26.45	0.52 (0.38–0.72)^*^	0.57 (0.41–0.79)^*^	0.61 (0.44–0.85)^*^

HR, hazards ratio.

Model 1 was adjusted for age and gender.

Model 2 was adjusted as model 1 plus *hukou*, body mass index (BMI), education, marriage, drinking, and smoking.

All 16 items were entered simultaneously in model 3.

^*^ means *P* < 0.05.

### 3.3. Stratified analysis of the association between balance ability and death risk

In the overweight and obese group and the non-overweight and obese group, for every second increase in balance, the risk of death in the population decreased by 16 and 7%, respectively (*HR* = 0.84,95% CI: 0.76–0.92; *HR* = 0.93, 95% CI: 0.86–0.99). For those persons with the balance ability <10 s as the control group, the risk of death of ≥10 s decreased by 55 and 35% in the overweight and obese and the non-overweight and obese, respectively (*HR* = 0.45,95% CI: 0.23–0.88; *HR* = 0.67, 95% CI: 0.46–0.97). There was no interaction between overweight and obesity and continuous balance ability (*HR* = 0.91, 95% CI: 0.81–1.02), as well as binary balance ability (*HR* = 0.70, 95% CI: 0.33–1.50) (Table 3).

**Table 3:** Cox risk proportional model of death risk among participants who were overweight and obese or not.

**Subgroup**	**Cases, no**	**Non-overweight and obesity (*****n*** = **10,256)**			**Cases, no**	**Overweight and obesity (*****n*** = **8,632)**			***HR* (95% CI) for interaction CI)**
		**Model 1**	**Model 2**	**Model 3**		**Model 1**	**Model 2**	**Model 3**	
**Balance ability**	1,0256	0.91 (0.85–0.97)^*^	0.92 (0.86–0.99)^*^	0.93 (0.86–0.99)^*^	8 632	0.83 (0.76–0.92)^*^	0.83 (0.75–0.91)^*^	0.84 (0.76–0.92)^*^	0.91 (−081–1.02)
**(continuous)**								
**Balance ability (binary)**									0.70 (0.33–1.50)
<10 s	104	1.00	1.00	1.00	52	1.00	1.00	1.00	
≥10 s	10,152	0.56 (0.38–0.80)^*^	0.62 (0.43–0.90)^*^	0.67 (0.46–0.97)^*^	8 580	0.43 (0.22–0.84)^*^	0.44 (0.23–0.86)^*^	0.45 (0.23–0.88)^*^	

Model 1 was adjusted for age and gender.

Model 2 was adjusted as model 1 plus *hukou*, education, marriage, drinking, and smoking (excluded BMI).

All 15 items were entered simultaneously in model 3.

^*^ means *P* < 0.05.

In the chronic disease group, for every second increase in balance ability, the risk of death decreased by 12% (*HR* = 0.88,95% CI: 0.82–0.94). For those people with a balance ability <10 s as the control group, the risk of death in the chronic disease group was reduced by 45% (*HR* = 0.55,95% CI: 0.38–0.79). There was no statistical significance in the predictive effect of continuous balance ability and binary variables on the survival of the non-chronic disease group (*HR* = 0.90,95% CI: 0.83–1.04; *HR* = 0.80,95% CI: 0.39–1.65). There was no interaction between chronic disease and continuous balance ability (*HR* = 0.95,95% CI: 0.84–1.08), as well as binary balance ability (*HR* = 0.76,95% CI: 0.34–1.68) (Table 4).

**Table 4:** Cox risk proportional model of death risk among participants with chronic disease or not.

**Subgroup**	**Number**	**Non-chronic disease status (*****n*** = **7,554)**			**Number**	**Chronic disease status (*****n*** = **11,334)**			***HR* (95% CI) for interaction CI)**
		**Model 1**	**Model 2**	**Model 3**		**Model 1**	**Model 2**	**Model 3**	
**Balance ability**	7,554	0.91 (0.82–1.02)	0.93 (0.83–1.04)	0.93 (0.83–1.04)	11 334	0.87 (0.81–0.93)^*^	0.86 (0.81–0.92)^*^	0.88 (0.82–0.94)^*^	0.95 (0.84–1.08)
**(continuous)**								
**Balance ability (binary)**									0.76 (0.34–0.168
<10 s	37	1.00	1.00	1.00	119	1.00	1.00	1.00	
≥10 s	7,517	0.70 (0.34–1.43)	0.79 (0.39–1.63)	0.80 (0.39–1.65)	11 215	0.47 (0.33–0.67)^*^	0.50 (0.35–0.73)^*^	0.55 (0.38–0.79)^*^	

Model 1 was adjusted for age and gender.

Model 2 was adjusted as model 1 plus *hukou*, body mass index (BMI), education, marriage, drinking, and smoking.

All 11 items were entered simultaneously in model 3 (excluded five chronic disease variables).

^*^ means *P* < 0.05.

Sensitivity analysis was performed on 18,888 middle-aged and older people without multiple interpolations, and the results are given in Table 5.

**Table 5:** Sensitivity analysis of the relationship between balance ability and death (original data).

**Outcomes**	***HR*** **(95% CI)**		
**Death**	**Model 1**	**Model 2**	**Model 3**
**Balance ability**	0.89 (0.84–0.94)^*^	0.90	0.91
**(continuous)**		(0.85–0.95)^*^	(0.86–0.96)^*^
**Balance ability**			
**(binary)**			
<10 s	1.00	1.00	1.00
≥10 s	0.53 (0.39–0.74)^*^	0.59	0.65
		(0.42–0.81)^*^	(0.46–0.90)^*^

Model 1 was adjusted for age and gender.

Model 2 was adjusted as model 1 plus *hukou*, body mass index (BMI), education, marriage, drinking, and smoking.

All 16 items were entered simultaneously in model 3.

^*^ means *P* < 0.05.

## 4. Discussions

We found that the risk of death decreased significantly, as the number of seconds of balance maintenance increased. After adjusting the covariates, this relationship still existed. Higher balance ability was independently related to the decrease in death risk in middle-aged and older people. The death density of middle-aged and older people whose balance ability <10 s was higher than that of those people with a balance ability ≥10 s. Consistent with a cohort study of 1,085 elderly people in Japan, standing balance predicts all-cause mortality and was independently associated with all-cause mortality (14). The poor balance ability of middle-aged and older people can be traced back to many possible reasons. With increasing age, the muscles of the vestibular-evoked responses will be delayed, resulting in a decline in balance ability (17). Multiple systems were affected by chronic inflammatory states and oxidative imbalances (18). When middle-aged and older people stood with their eyes open, the force area of the feet mainly depended on the transmission information of the plantar proprioception and the muscle strength of the lower limbs to maintain the posture (19). At this time, it is more likely to have problems such as reduced information transmission efficiency of the plantar mechanoreceptor, low utilization rate, flat proprioceptive information, and weak relative strength, which finally lead to a decline in balance ability (20).

The present study found that chronic disease, overweight, and obesity had no significant effect on the association between balance ability and death. A total of 152 studies found that being overweight or obese were associated with plantar sensitivity, lower sensitivity, and decreased postural stability observed in obese people (21). Balance control impairment in individuals with obesity may be caused by larger balance motor commands' variability (22). However, this association between balance ability and eventual death risk was insignificant. The present study did not find the interaction, even though chronic kidney diseases, cardiac diseases, cancer, or some other chronic diseases may lead to premature death (23–25). After calculation, the two confounders had no significant moderating effect on the association between balance ability and death risk. The inconsistency of interactions and subgroups may be due to the relatively small sample size. It is worth mentioning that early resistance training can improve balance ability and physical function in the elderly (26).

Our study supplements further the evidence of the relationship between balance ability and death in middle-aged and older people based on the Chinese population. It is a community-based prospective study with good national representation. Stratification of factors, such as chronic disease, overweight, and obesity, was considered at the design stage, and potential confounding factors were adjusted to reinforce the existing evidence. However, the limitations of this study are that, first of all, the grouping of balance ability is uneven and does not consider the balance ability beyond 10 s may be achieved. Second, balance ability is a single body index, which needs to be combined with different measurements of body function or can improve the predictive ability of death risk. Third, all-cause deaths include deaths caused by external forces not directly related to health, such as car accidents, and there may be underreporting. Finally, overweight and death were combined in the subgroup analysis, which weakened the effect of balance ability on death in a single BMI status.

In summary, the balance ability of middle-aged and older people was associated with the risk of all-cause death, and this association did not decrease with the presence or absence of chronic diseases, overweight, and obesity. The measurement of balance ability can be included as a routine examination method. When the physical examination shows that the balance ability of middle-aged and older people is significantly weakened, the reasons that may cause the balance ability to decline should be screened. For middle-aged and older people with poor balance ability and obvious clinical symptoms, it may be appropriate to increase the balance ability training to intervene and avert a fall.

## Data availability statement

Publicly available datasets were analyzed in this study. This data can be found here: https://charls.pku.edu.cn/en/.

## Ethics statement

The studies involving human participants were reviewed and approved by Peking University (IRB 00001052-11014). The patients/participants provided their written informed consent to participate in this study. Written informed consent was obtained from the individual(s) for the publication of any potentially identifiable images or data included in this article.

## Author contributions

KHX planned the study, supervised the data analysis, and wrote the paper. XH performed all statistical analyses and contributed to revising the paper. XHH helped find the source and analysis method. All authors have read and approved the manuscript and they have no conflicts of interest.
